# Supplementary material for: Seroprevalence and risk factors associated with bovine tuberculosis in cattle in Eastern Bhutan
Source: PLoS Negl Trop Dis. 2024 May 28;18(5):e0012223. doi: 10.1371/journal.pntd.0012223 (PMC11161125; doi:10.1371/journal.pntd.0012223)
Supplement: S1 Table — (DOCX) [file pntd.0012223.s001.docx]

Supplementary table 1: Factors associated with bovine TB in Eastern Bhutan

|  | **Proportion** | | **Unadjusted** | | | **Adjusted** | | |
| --- | --- | --- | --- | --- | --- | --- | --- | --- |
| **Characteristic** | **Negative**, N = 946^1^ | **Positive**, N = 25^1^ | **OR**^2^ | **95% CI**^2^ | **p-value** | **OR**^2^ | **95% CI**^2^ | **p-value** |
| **District** |  |  |  |  |  |  |  |  |
| Mongar | 273 (29%) | 14 (56%) | 1.87 | 0.66, 6.70 | 0.3 |  |  |  |
| Trashigang | 246 (26%) | 4 (16%) | 0.59 | 0.14, 2.54 | 0.5 |  |  |  |
| Lhuentse | 146 (15%) | 4 (16%) | — | — |  |  |  |  |
| Trashiyangtse | 109 (12%) | 1 (4.0%) | 0.33 | 0.02, 2.30 | 0.3 |  |  |  |
| Samdrup Jongkhar | 90 (9.5%) | 1 (4.0%) | 0.41 | 0.02, 2.79 | 0.4 |  |  |  |
| Pemagatshel | 82 (8.7%) | 1 (4.0%) | 0.45 | 0.02, 3.07 | 0.5 |  |  |  |
| **Cow breed** |  |  |  |  |  |  |  |  |
| Crossbred | 679 (72%) | 18 (72%) | — | — |  |  |  |  |
| Indigenous | 267 (28%) | 7 (28%) | 0.99 | 0.38, 2.30 | >0.9 |  |  |  |
| **Sex** |  |  |  |  |  |  |  |  |
| F | 875 (92%) | 24 (96%) | — | — |  |  |  |  |
| M | 71 (7.5%) | 1 (4.0%) | 0.51 | 0.03, 2.49 | 0.5 |  |  |  |
| **Age of cattle** |  |  |  |  |  |  |  |  |
| 4-8 years | 589 (62%) | 18 (72%) | 3.59 | 1.03, 22.7 | 0.088 | 3.75 | 1.07, 23.7 | 0.078 |
| <4 years | 235 (25%) | 2 (8.0%) | — | — |  |  |  |  |
| >8 years | 122 (13%) | 5 (20%) | 4.82 | 1.02, 34.0 | 0.063 | 5.12 | 1.08, 36.2 | 0.054 |
| BCS |  |  |  |  |  |  |  |  |
| 3-4 | 645 (68%) | 20 (80%) | 1,319,218 | 0.00, NA | >0.9 |  |  |  |
| 1-2 | 279 (29%) | 5 (20%) | 762,451 | 0.00, NA | >0.9 |  |  |  |
| >4 | 22 (2.3%) | 0 (0%) | — | — |  |  |  |  |
| **Cough** | 194 (21%) | 8 (32%) |  |  |  |  |  |  |
| No |  |  | — | — |  |  |  |  |
| Yes |  |  | 1.82 | 0.73, 4.17 | 0.2 | 2.21 | 0.87, 5.18 | 0.077 |
| **Respiratory distress** | 99 (10%) | 3 (12%) |  |  |  |  |  |  |
| No |  |  | — | — |  |  |  |  |
| Yes |  |  | 1.17 | 0.27, 3.44 | 0.8 |  |  |  |
| **Nasal discharge** | 77 (8.1%) | 1 (4.0%) |  |  |  |  |  |  |
| No |  |  | — | — |  |  |  |  |
| Yes |  |  | 0.47 | 0.03, 2.27 | 0.5 | 0.28 | 0.02, 1.47 | 0.2 |
| **Mastitis** | 72 (7.6%) | 3 (12%) |  |  |  |  |  |  |
| No |  |  | — | — |  |  |  |  |
| Yes |  |  | 1.66 | 0.39, 4.92 | 0.4 |  |  |  |
| **History of abortion** | 55 (5.8%) | 3 (12%) |  |  |  |  |  |  |
| No |  |  | — | — |  |  |  |  |
| Yes |  |  | 2.21 | 0.51, 6.63 | 0.2 |  |  |  |
| **Fetal membrane retention** | 41 (4.3%) | 2 (8.0%) |  |  |  |  |  |  |
| No |  |  | — | — |  |  |  |  |
| Yes |  |  | 1.92 | 0.30, 6.80 | 0.4 |  |  |  |
| **Source of animal** |  |  |  |  |  |  |  |  |
| Farm born | 920 (97%) | 23 (92%) | — | — |  |  |  |  |
| Imported | 26 (2.7%) | 2 (8.0%) | 3.08 | 0.48, 11.2 | 0.14 |  |  |  |
| ^1^n (%) | | | | | | | | |
| ^2^OR = Odds Ratio, CI = Confidence Interval | | | | | | | | |

BCS (Body condition score)
