# Supplementary material for: Seroprevalence and risk factors associated with bovine tuberculosis in cattle in Eastern Bhutan
Source: PLoS Negl Trop Dis. 2024 May 28;18(5):e0012223. doi: 10.1371/journal.pntd.0012223 (PMC11161125; doi:10.1371/journal.pntd.0012223)
Supplement: Attachment — Submitted filename: Reviewer comment.docx [file pntd.0012223.s003.docx]

**Response to reviewers’ comment**

Methods


-Are the objectives of the study clearly articulated with a clear testable hypothesis stated?

-Is the study design appropriate to address the stated objectives?

-Is the population clearly described and appropriate for the hypothesis being tested?

-Is the sample size sufficient to ensure adequate power to address the hypothesis being tested?

-Were correct statistical analysis used to support conclusions?

-Are there concerns about ethical or regulatory requirements being met?


Reviewer #1: Yes the objectives of the study are clear, design is appropriate, sample size is sufficient, statistical analysis were adequate and ethical statement is being presented

Answer: Thank you

Reviewer #2: The Material and Methods section must be re-structured. The population is not clearly described, data on the population is missing, and it is highly relevant to make inferences and to understand how prevalence are estimated.
It is long and reiterative. Some data may be omitted or moved to Supplementary material (i.e., laboratory analysis).

Answer: We have made changes as commented by the reviewers.

Results


-Does the analysis presented match the analysis plan?

-Are the results clearly and completely presented?

-Are the figures (Tables, Images) of sufficient quality for clarity?


Reviewer #1: Yes the analysis, results and figures are appropriate

Reviewer #2: The results section should also be re-structured. Descriptive data should be included. Results of the logistic regression analysis (univariable and multivariable) are missing. The order of the Tables should be changed.

--------------------

Conclusions


-Are the conclusions supported by the data presented?

-Are the limitations of analysis clearly described?

-Do the authors discuss how these data can be helpful to advance our understanding of the topic under study?

-Is public health relevance addressed?


Reviewer #1: Yes the conclusions were discussed and presented. The public health significance can be elaborated

Reviewer #2: Conclusions should be expanded and modified in line with the comments suggested, with a more detailed explanation of the relevance for public health management in the country and worldwide. One point requiring additional work, is being more explicit in what the impact of this work is for disease control, policy makers, and researchers.

--------------------

Editorial and Data Presentation Modifications?


Use this section for editorial suggestions as well as relatively minor modifications of existing data that would enhance clarity. If the only modifications needed are minor and/or editorial, you may wish to recommend “Minor Revision” or “Accept”.

Reviewer #1: Minor revision with some more discussion on the ELISA idexx kit whether it is a OIE recommended kit with some relevant publications based on this kit. There can be a discussion why author preferred B cell medicated diagnostic rather than T cell medicated. The public health significance of the study needs to be presented. Why the sero prevalence came less need to be elaborate whether hilly terrain or herd size or any other factor they consider to be important.

Answer: Thank you for the suggestion. We have rewritten the discussion and incorporated all the necessary information in the discussion and the conclusion.

Reviewer #2: (No Response)

--------------------

Summary and General Comments


Use this section to provide overall comments, discuss strengths/weaknesses of the study, novelty, significance, general execution and scholarship. You may also include additional comments for the author, including concerns about dual publication, research ethics, or publication ethics. If requesting major revision, please articulate the new experiments that are needed.

Reviewer #1: Bovine tuberculosis-being am important animal and public health disease need to be studied. And as in Bhutan no previous studies of such is presented so it is important that such work should be carried out. Further tuberculin test based studies are also being encouraged by authors in there next project. The consumption of raw milk among public need to be also studied

Answer: Thank you for the comments on importance of the study.

Reviewer #2: The authors estimate the seroprevalence of bovine tuberculosis in Bhutan. This is the first study conducted in the country, so it provides a valuable information to get insights into the epidemiological situation of the disease in the area, surrounded by high prevalence countries. Overall, the conclusions of this article should be analyzed with caution. It is always complicated to estimate the prevalence of bovine tuberculosis in the absence of a gold standard, specially by using the ELISA test. It should be noted that this may be due to logistic reasons, which should be clearly mentioned along with the implications of it. I would like the authors to discuss a little more about the possible underestimation of the true prevalence induced here using ELISA test.
Thank you and congratulations for this very necessary and interesting work. Overall, this paper would benefit from some major revision, so I suggest some corrections and recommendations that may make it clearer and easier to read.

Answer: Thank you for the comments. We have incorporated the changes and highlighted as mentioned below.

ABSTRACT
- I suggest adding a statement that the study was conducted in a region of Bhutan and did not include samples from across the country.

Answer: We have added the sentences that specifies where the study was conducted.

Line number 28-29: This study aimed to determine the seroprevalence of bTB in cattle in six districts of eastern Bhutan.

- Lines 27-28: “Although bTB is a zoonotic disease…”. This was already stated. Please avoid reiterating.

Answer: The sentences has been deleted.

- Line 31: “serum samples”?

Answer: we have rephrased the sentences.

Line number 29-31: A two stage probability proportional to size (PPS) sampling strategy was used to determine the number of animals from which serum samples needed to be collected in each district and sub-district.

- Is it possible to include a statement regarding the cattle census of which you randomly select samples?

Answer: We have added a statement elaborating on this.

Line number 31-32: All the farms and the cattle for sampling were selected randomly from the annual livestock census 2020.

- Lines 33-34: Please include information on the nature of the risk factors evaluated.
Answer: we have added the factors evaluated.

Line number 33-36: Logistic regression models were constructed to assess the influence of various individual animal and environmental risk factors (breed, age, sex, source of animal, body condition scores of animals, respiratory system status) associated with sero-positivity in animals.

INTRODUCTION
- Line 43: Although M. bovis represents the main causative agent of bTB, it may be caused by members of the Mycobacterium tuberculosis complex. Please rephrase.

Answer: we have rephrased the sentences.

Line number 66-67: Bovine tuberculosis (bTB) is a chronic zoonotic infection caused by Mycobacterium tuberculosis complex, primarily by *Mycobacterium bovis*.

- Lines 46-47: I suggest including a statement regarding other important aspects of the disease, such as latency and anergy, which add extra complexity to disease control.

Answer: We have added additional information on it.

Line number 70-72: The disease mainly infects lungs and other organs in later stages, with progression occurring over a period ranging from months to years in some cases (1). In addition, latent infections and anergic reactors pose challenges in detecting and preventing bTB (2).

- Line 50: Please change from “accounts” to “accounted”, as these are values from 2016. Additionally, could authors provide more up-to-date estimates?

Answer: We have changed the word. However, it terms of the estimates, this is the latest data that we could access.

Line number 77-78: Besides, the disease accounted for 10% of human TB cases with 147,000 new cases of zoonotic TB in humans and 12,500 deaths in 2016 alone (6).

- Line 52: Please add “may” before “get infected”. Are these contaminated aerosols derived from the close contact with infected animals? Please clarify.

Answer: The sentences has been rephrased.

Line number 79-80: Humans may contract bTB by consuming raw milk or by inhaling contaminated aerosols when in close contact with infected animals (7,8).

- Lines 56-57: Please revise grammar and rephrase.

Answer: The sentences has been rephrased.

Line number 83-84: Bovine tuberculosis is found globally, with the highest occurrence observed in countries across Africa and Asia (9).

- Lines 57-59: In which countries? Could you provide further information that support this data? Any examples? In fact, there are several countries where, despite the efforts invested in bTB eradication through control programs (including developed countries), the disease is still endemic, and not negligible levels. Please correct.

Answer: The additional information has been added.

Line number 87-89: However, the disease remains endemic and uncontrolled in Africa, Asia, Latin America and most countries in the Middle East (12-15) posing great economic and public health threat.

- Lines 61-72: Please include references that support this statement (Lines 61-62). Additionally, there are some important risk factors missing (i.e., contact with other potential wild reservoirs, use of pastures leading to potential contact with other reservoirs -domestic and/or wildlife-, prevalence of the area,…). Please expand your explanation regarding this.

Answer: It has been updated.

Line number 92-94: The prevalence of bTB is influenced by risk factors such as individual animal level, herd level, and wildlife reservoirs (19). Individual risk factors include age, breed, sex, body condition, immune status, and genetic resistance of the animal (20, 21).

- Lines 69-70: I suggest adding other reference where the differences in the risk of bTB based on herd size are more evident (i.e., Conlan AJK, McKinley TJ, Karolemeas K, Pollock EB, Goodchild AV, et al. (2012) Estimating the Hidden Burden of Bovine Tuberculosis in Great Britain. PLoS Comput Biol 8(10): e1002730)

Answer: Thank you for the comment. We have added the references.

Line number 102-103: Moreover, the risk of transmission also increases with the increase in herd size (25).

- Line 74: Please correct to “Single intradermal tuberculin (SIT) test”. Additionally, I suggest a adding some clarification of the limitations of the currently available diagnostic tests in terms of performance, as it is mentioned in Lines 179-181 (please see de la Rua et al., 2016 and Nunez-Garcia et al., 2018).

Answer: It has been corrected. We have addressed the limitations in responses to these questions.

Line number 106-107: Single intradermal tuberculin test (SIT) with a sensitivity of 92% and specificity of 89% (26) is commonly used test to identify positive reactors.

- Lines 77-78: I would not define it as “easy to apply” rather than ELISA provides a more standardized interpretation (i.e., it may be less subjective). Additionally, what about the sensitivity? Although it may be further discussed, it would be good to provide some context regarding why the ELISA is used in this epidemiological scenario.

Answer: We have rephrased the sentences. The sensitivity and specificity are discussed in the method section and choice of ELISA over other methods are discussed in the discussion section.

Line number 109-113: SIT, CIT and IFN- γ tests cell mediated immune response. SIT and CIT requires two filed visits, restrain of animal and injection of bTB antigen. IFN- γ requires time limited cell sensitisation, incubation period and blood sample collection. In contrast, the ELISA is logistically easier to perform and provides standardised interpretation and exhibits higher specificity (96%) compared to the SIT (14).

Line number 161-163: The bTB testing was performed using WOAH approved ELISA kit (Antibody Test Kit from IDEXX (35) with a diagnostic sensitivity of 65% and specificity of 98% (35).

Line number 285-301: It is also important to acknowledge that the lower seroprevalence detected in this study may be influenced by the choice of test method employed. Mycobacterium bovis antibody ELISA test kit used in this study has a sensitivity of 65%, notably lower than the intradermal tuberculin test commonly used for bTB detection (43). Furthermore, the sensitivity of ELISA test kit has been reported to be considerably lower than standard intradermal skin test (2) with field-level sensitivity ranging from 4-9% compared to the intradermal test and 9-14% compared to post-mortem techniques (McCallan et al., 2021). However, the sensitivity of ELISA kit increases to 66-85% when tested on samples from post-skin test in the same cohort (44). Use of only one test with lower sensitivity in a tuberculin unsensitised population may result in missed bTB positive cases detection. Additionally, antibody tests like ELISA are ineffective in detecting sub-clinical infections, as their sensitivity increases only when infections progress and higher antibody levels are produced (45). However, the ELISA test specificity of 98% that we have used is comparable to the gold standard culture test (99.1%), minimizing false positives (46). Although the ELISA test is recommended for use alongside intradermal tuberculin tests for screening purposes, the latter was not utilized in this study to avoid introducing TB antigen into anergic cattle herds (43), and due to logistical constraints. Therefore, screening animals using antibody-detecting test kits was deemed the most feasible approach for determining baseline seroprevalence in this study.

- Line 80: but is it so because there is no bTB or rather it has not been tested yet? Any suspicions regarding bTB circulation in cattle population?

Answer: Previously, a surveillance in the peri-urban areas of Thimphu (capital city) was performed by Tenzin and team (*Bovine tuberculosis survey in peri-urban dairy farm, Thimphu. Jpurnal of Renewable Natural Resources Bhutan*) with sample size of 66 cattle. No cases of btb were detected in that study.

- Lines 81-85: Please include the year/range of years of these prevalence estimates, to provide a temporal context.

Answer: The year of study has been added.

Line number 115-118: In India, the bTB prevalence varied from 40% in Assam in 2014 (28) to over 25% in West Bengal in 2017 (29), which shares a border with Bhutan. A recent study in Bangladesh in 2019 revealed an alarming overall bTB prevalence in the region, reported at 45.6% (17).

- 86-94: Just preventive measures or is it control and prevention measures? I suggest expanding the background and hypotheses of this study, maybe it would ease the reader to start with the statement presented in lines 88-91. Please clarify and rephrase.

Answer: The statement has been rephrased as mentioned below.

Line number 118-125: Bhutan has recently imported live cross-bred cattle from India to enrich its genetic diversity, inadvertently raising the risk of introducing infectious diseases such as bTB (27). Furthermore, there has been a shift in Bhutan's cattle rearing practices from extensive free grazing to more intensive indoor stall-feeding systems (30), which may contribute to increased transmission of bTB. Therefore, it is imperative to assess the disease status among cattle in Bhutan to institute appropriate control and preventive measures. Accordingly, this study aimed to determine the seroprevalence of bovine tuberculosis in cattle in eastern Bhutan and identify the associated risk factors for seropositivity.

MATERIAL AND METHODS
Overall, it would ease the reader to re-structure this section, maybe by including a lower number of subheadings (i.e., merge and re-structure sample size, sampling and sample collection subheadings) and specially by adding an expanded prior descriptive analysis of the cattle population. What is the population of which you take the sample? Proportion of herds out of the total cattle population in the region? Overall animals per herd? The sampling method is poorly presented. Was the sampling performed per district? How many herds per region/district? Additionally, the objectives should be clearly mentioned, along with the population under study, the outcome, and the statistical models applied.

Answer: We have grouped the headings and decreased the number of sub-headings. And we have also added additional information as advised. We have rewritten the sampling strategy. The number of samples collected from each district is added in table 2.

Line number 158-160: During the study period, six eastern districts have a cattle population of 96,581 (Mongar district: 26,977; Trashigang: 25,532; Lhuentse: 13,933; Samdrup Jongkhar: 12,191; Tashiyangtse: 10,150; Pemagatshel: 7,79) (32).

Line number 167-175: A two-stage probability proportional to size (PPS) sampling strategy was used to determine the number of samples to be collected from each district and sub-district. The calculation was preformed using Microsoft Excel (Microsoft Excel, Redmond USA) with a district cattle data used for selection (32). The number of samples to be collected from each district was determined based on the cattle population of that district. Similarly, the cattle population of each sub district and the total sample of that district was used to determine the total samples to be collected from each sub district. All household owning cattle in each sub-district were assigned a random number to select the households. In cases where households had more than one cattle, the animal for sampling was chosen using simple random sampling to prevent any potential selection bias in the study.

- Lines 97-99: Do authors mean that Bhutan is subdivided into 4 regions that include 20 districts? One RLDC in the East, West, East Central and West Central? Please rephrase. Additionally, I suggest using another verb rather than “cater”.

Answer: We have rewritten the paragraph. For veterinary and livestock services, 20 districts of Bhutan are clustered into four regions namely Regional Livestock Development Centre (RLDC) in the East, West, East Central and West Central. This is displayed in the Bhutan map (Figure 1).

Line number 128-131: Bhutan is administratively subdivided into 20 districts. Its veterinary and livestock services are organized into four regions, each overseen by a Regional Livestock Development Centre (RLDC) located in the East, West, East Central, and West Central at the time of conducting this study in 2021.

- Line 99: Authors are assuming that the study was conducted in the eastern RLDC. Please add a clarification regarding the study area. I suggest something similar “The study was conducted in the East region, where the eastern RLDC provide veterinary services to six districts:…”

Answer: It has been rephrased as advised.

Line number 131-133: This study was conducted within the eastern RLDC region, which provides services to six districts: Trashigang, Trashiyangtse, Lhuentse, Pema Gatshel, Mongar, and Samdrup Jongkhar (Figure 1).

- Line 103: Please correct “owing to” to “due to”.

Answer: The sentences has been rephrased.

Line number 135-136: Due to financial and logistical constraints, the study's scope was limited to this specific region.

- Line 102: Please provide information on cattle census (i.e, number of cattle herds in the region, in each district), the population under study and from which you take the sample.

Answer: We have added this information. And moreover, we have added additional information in table 1.

Line number 158-160: During the study period, six eastern districts have a cattle population of 96,581 (Mongar district: 26,977; Trashigang: 25,532; Lhuentse: 13,933; Samdrup Jongkhar: 12,191; Tashiyangtse: 10,150; Pemagatshel: 7,79) (32).

- Lines 105-110: Far too long a sentence. Please rephrase.

Answer: The sentences has been rephrased and shortened.

Line number 144-147: Different cattle rearing systems are practiced based on the area's topography. In the higher altitude regions such as Trashigang, Trashiyangtse, and Lhuentse, a transhumant system is practiced, wherein cattle migrate seasonally. In the lower altitude areas like Mongar, Pemagatshel, and Samdrup Jongkhar, a sedentary system is practiced (31).

- Lines 110-111: Do authors mean that herds located in higher altitudes tend to practice stall-feeding, which are mainly crossbred dairy cattle? Please rephrase as it is confusing.

Answer: We have rephrased the sentences.

Line number 147-149: In the sedentary system, indigenous cattle are allowed to freely graze on shared pastures and watering points, while high yielding crossbred dairy cattle are stall-fed (30).

- Lines 111-113: Where? Across the region? In high altitudes? Please check grammar and rephrase. I suggest using the term shared pastures and watering points.

Answer: We have rephrased the sentences.

Line number 147-149: In the sedentary system, indigenous cattle are allowed to freely graze on shared pastures and watering points, while high yielding crossbred dairy cattle are stall-fed (30).

- Lines 116-117: This mention to the study area should be stated before, as suggested earlier. Additionally, I suggest “to determine the seroprevalence of bTB in cattle in Eastern Bhutan a cross-sectional study was conducted”.

Answers: It is rephrased as advised.

Line number 151-152: This was a cross-sectional study conducted to determine the seroprevalence of bTB, from March 2021 to November 2021.

- Line 118: With source of animal, did you mean herd of origin?

Answer: We meant if the animal was farm born or imported.

Line number 152-153: Data on animal age, sex, breed, and source of cattle (imported or farm born) were collected using a semi-structured questionnaire.

- Lines 122-129: please expand this section as stated before. What proportion of the total cattle herds/animals does 863 represent? Please expand the explanation on the coverage of the study samples (numerator) with respect to the cattle population of the region/district (denominator).

Answer: We determined the sample size of 863 based on criteria of the study. We have elaborated as follows.

Line number 163-166: Due to the absence of bTB prevalence data in Bhutan or in eastern Bhutan, a prevalence of 50% was assumed to maximize the sample size, requiring 863 cattle to be sampled from a total of 96,581 for this study. Ultimately, 971 samples were collected to increase the power of the study and to account for potential damage or loss of samples.

- Line 128: Please change to “Mycobacterium bovis”.

Answer: We have rewritten the paragraph.

- Line 134: Please provide an extended descriptive analysis of the population, maybe extracted from this reference? Please include these descriptive at the beginning of the section and combine with information provided in Lines 132-141.

Answer: We have added the information.

Line number 158-160: During the study period, six eastern districts have a cattle population of 96,581 (Mongar district: 26,977; Trashigang: 25,532; Lhuentse: 13,933; Samdrup Jongkhar: 12,191; Tashiyangtse: 10,150; Pemagatshel: 7,79) (32).

- Line 144: I suggest adding a mention earlier in Material and Methods regarding the year under study (2021, maybe in my comment of Lines 116-117?).

Answer: The sentences is rephrased and now the year of study is noted in the study design.

Line number 151-152: This was a cross-sectional study conducted to determine the seroprevalence of bTB, from March 2021 to November 2021.

- Lines 146-147: Please move “was collected” at the end of the sentence.

Answer: The sentence is changed

Line number 180-181: Approximately 6-7 mL of blood via jugular venepuncture into plain vacutainers (BD, India) was collected.

- Lines 157-171: Material and Methods is a very long section. Have authors considered moving “Laboratory analysis” section to Supplementary material? Is it relevant to include all this information in the main manuscript?

Answer: It has been deleted from paragraph as it was based on manufacturers protocol.

- Line 175: In line with my previous comments, which descriptive data?

Answer: The sentences is rephrased as below.

Line number 197-198: The demographic and clinical data are presented in frequency and proportions.

- Lines 178-179: Why were these values used? Could you provide any reference supporting these estimates?

Answer: These values were used as per the manufacturer’s instructions and the relevant references has been cited.

Line number 199-201: The diagnostic sensitivity (Se) and the specificity (Sp) of bTB testing ELISA kit that was used for this study was 65% and 98%, respectively (35).

- Lines 183-184: I would avoid using the term “known sensitivity and specificity of the test” as these are just estimates usually surrounded by a wide uncertainty. Please rephrase.

Answer: The sentences has been rephrased.

Line number 205-206: Using the estimated sensitivity and specificity of the test,

- Line 185: Please add reference and correct (Rogan and Gladen, 1978) same for line 190.

Answer: The references has been added for both.

- Line 200: Age was categorized into three categories. Are these terciles? How was the distribution of data?

Answer: The age was not normally distributed. To explore for factors associated with positivity, it was categorized into three groups.

- Line 204: Please do not use abbreviations at the beginning of the sentence.

Answer: The sentences has been joined to avoid beginning with abbreviations.

Line number 223-225: A five-point scale was used to estimate the body condition score (BCS) of the cattle (42), with categories of 1-2 for thin, 3-4 for average, and >4 for fat cattle.

- Lines 211-212: Results, please remove from Material and Methods.

Answer: It has been removed and added in the result section.

Line number 256-259: We also fitted the demographic and clinical data to multiple logistic regression and analysed for confounding factors using backward elimination (Supplementary table 1). However, none of the variables were significant, it is not shown here.

- Lines 214-217: Maybe this section can be presented at the end of the manuscript.

Answer: It has been moved to section after conclusion.

RESULTS
- Overall, in this section, I suggest adding the “n” next to the % values (n = XX).
- Line 221-222: Please add “The maximum number of” before samples. Additionally, information on the cattle population per district is absent.

Answer: We have added percentage and number after all the data.

Line number 231-232: The maximum number of samples were collected from Mongar district considering its large cattle population.

- Lines 222-224: Actually, and in my opinion, Table 2 includes more descriptive data on the population under study than Table 1. Table 2 includes data on the estimated seroprevalence.

Answer: We have changed the table numbers and its position.

- Line 224: Please expand the title of the Table with the information included in it (i.e., number of samples, number of positive samples, etc.). Additionally, information in this table is scarce. It would be very useful to provide data on the number of samples per sub-district, along with the cattle census/herds in them (again, number of herds, number of tested herds, number of samples per herd, here in the Table or elsewhere). Otherwise, what is the point of including the Sub-districts column in the Table?

Answer: We have edited the table and added information in table 2.

- Lines 227-228: Please provide data on the age distribution (i.e., median/mean age, interquartile range), and if a statistical test was used to evaluate these differences between young, adult and old and the outcome variable it should be stated, if not, please include it.

Answer: It has been added.

Line number 241-243: The median age of animal was 5 years (IQR 3 years), with significant differences observed between male and female cow (Wilcoxon test p-value <0.001). There were no significant differences in bTB cases by age groups (p-value 0.283).

- Lines 228-229: Please check grammar.

Answer: We have rephrased the sentences.

Line number 243-244: Of the total, 52.3% (n=513) were farm born while 2.8% of them being imported from India

- Line 238: Please add %.

Answer: It has been added.

Line number 53: The apparent seroprevalence of bTB was 2.6% (n=25) (95% CI:1.6 - 3.6).

- Lines 238-242: It would be nice to include a graph with the map of the regions colored by the estimated seroprevalence.

Answer: We have edited and included additional information in the map (Figure 2).

DISCUSSION
- Lines 257-275: Please provide the reference and the CI of this estimate. Please discuss why you chose this Se estimate and what are the implications of using this/other estimates. Overall, I suggest a more expanded discussion on the major limitation of this study, which is the use of the ELISA to estimate the prevalence of bTB in the country, compared to other tests with higher Se. Please be more specific when you discuss results obtained here compared to other studies and countries.

Answer: The limitations of the study has been elaborated in the end of discussion.

Line number 285-314: It is also important to acknowledge that the lower seroprevalence detected in this study may be influenced by the choice of test method employed. Mycobacterium bovis antibody ELISA test kit used in this study has a sensitivity of 65% (59.7% - 69.5%), notably lower than the intradermal tuberculin test commonly used for bTB detection (44). Furthermore, the sensitivity of ELISA test kit has been reported to be considerably lower than standard intradermal skin test (2) with field-level sensitivity ranging from 4-9% compared to the intradermal test and 9-14% compared to post-mortem techniques (2). However, the sensitivity of ELISA kit increases to 66-85% when tested on samples from post-skin test in the same cohort (45). Use of only one test with lower sensitivity in a tuberculin unsensitised population may result in missed bTB positive cases detection. Additionally, antibody tests like ELISA are ineffective in detecting sub-clinical infections, as their sensitivity increases only when infections progress and higher antibody levels are produced (46). However, the ELISA test specificity of 98% that we have used is comparable to the gold standard culture test (99.1%), minimizing false positives (47). Although the ELISA test is recommended for use alongside intradermal tuberculin tests for screening purposes, the latter was not utilized in this study to avoid introducing TB antigen into anergic cattle herds (44), and due to logistical constraints. Therefore, screening animals using antibody-detecting test kits was deemed the most feasible approach for determining baseline seroprevalence in this study.

Moreover, use of the ELISA test for bovine tuberculosis (bTB) that have a sensitivity of 65%, indicates that approximately 35% of true seropositive cases may be missed. This presents a risk of false negatives, where infected cattle may go undetected due to factors such as infection stage and antibody level fluctuations. As a results, there is a potential for underestimating bTB seroprevalence when mainly relying on ELISA testing. Additionally, the test's specificity of 98% implies a 2% risk of false positives, attributing negative cases as positive. False positives may arise from cross-reactivity or testing errors. In summary, while ELISA testing provides valuable insights, confirming seropositive results with complementary methods, such as intradermal tuberculin tests is necessary for ensuring accurate diagnosis and surveillance efforts. This approach helps to minimize the risk of misinterpretation of results and enhances overall detection accuracy. This is the main limitation of the ELISA test and complement it with other diagnostic methods to improve the accuracy of bTB detection and surveillance efforts. Therefore, it is important to acknowledge this limitation of the ELISA test that we have used in this study.

- Lines 277-278: Please be careful with this statement, in line with my previous comments.

Answer: The paragraph has been rewritten.

Line number 315-321: The low seroprevalence of bovine tuberculosis (bTB) observed in our study should not be directly compared with the high detection rates reported in other studies. For example, in Assam State, India, which shares a border with one of the districts (Samdrup Jongkhar) of eastern Bhutan, a prevalence of 28% was reported, with tuberculin tests conducted on suspected bTB cattle (28). Similarly, in Nepal, bTB prevalence ranged from 11% (47) using single intradermal tuberculin tests to 45.7% (48) using tuberculosis antibody test kits, with again samples collected from suspected bTB cattle.

- Lines 289-291: Consider that, due to the lack of performance of bTB diagnostic tests, infected but not detected cattle may be moved between farms. Please add some statement regarding this issue.

Answer: As suggested, this has been included.

Line number 292-296: Use of only one test with lower sensitivity in a tuberculin unsensitised population may result in missed bTB positive cases detection. Additionally, antibody tests like ELISA are ineffective in detecting sub-clinical infections, as their sensitivity increases only when infections progress and higher antibody levels are produced (46).

- Lines 291-292: I suggest starting with this issue, it may be the main reason here.

Answer: The line has been rephrased and brought to start of paragraph.

Line number 285-286: It is also important to acknowledge that the lower seroprevalence detected in this study may be influenced by the choice of test method employed.

- Line 293: Please change to Mycobacterium bovis.

Answer: The paragraph has been rewritten and hence deleted.

- Lines 297-298: I am not totally comfortable with this statement at all. Please correct.

Answer: The sentences has been rephrased.

Line number 297-300: Although the ELISA test is recommended for use alongside intradermal tuberculin tests for screening purposes, the latter was not utilized in this study to avoid introducing TB antigen into naive cattle herds (44), and due to logistical constraints.

- Line 301: Please change to “A higher number of positive cases…”

Answer: The sentences has been changed.

Line number 328-330: A higher number of seropositive cases were recorded in crossbred cattle (18/25) compared to indigenous breeds, which could be due to the genetic composition of the indigenous breed that may have higher resistance to infection (14).

- Line 303: Please use “may have” instead of “has”.

Answer: It is changed.

Line number 328-330: A higher number of seropositive cases were recorded in crossbred cattle (18/25) compared to indigenous breeds, which could be due to the genetic composition of the indigenous breed that may have higher resistance to infection (14).

- Line 304: Please delete “than male”.

Answer: It is deleted. Thank you for pointing it out.

- Line 308: Please add “a” before “higher number”.

Answer: We have rewritten the discussion section.

- Lines 315-326: Newly infected animals may become undetectable or have a poor response to the in vivo tests (see Alvarez et al., 2014; Barlow et al., 1997; Conlan et al., 2012), and these may be being missed here. As bTB progresses, there is a shift towards the development of humoral responses (which is measured in this study). It should be noted that infected animals with clinical signs are mainly those in more advanced stages of the disease. In line with my previous comments, what about the possibility that infected animals in the first stages of the infection may be missed because of the limited sensitivity of the test?
- Could you please discuss whether these estimates might be extrapolated to the whole country? Why?

Answer: We have rewritten the discussion section.

CONCLUSION
- I suggest a more specific conclusion after the current review.

Answer: The conclusion has been rewritten

# Line number 365-391:

Based on the findings and conclusions of this study, we recommend the following:

- Implement comprehensive surveillance programs using more sensitive diagnostic tests to accurately determine the prevalence and distribution of bovine tuberculosis (bTB) in Bhutan. This includes regular testing of cattle populations across different regions of the country. A risk-based surveillance strategy focusing on cattle along the international border, imported animals, commercial dairy farms and those showing clinical signs suggestive of bTB may be prioritized to optimize surveillance efforts and manage costs effectively.
- Invest in training programs for veterinary professionals and laboratory personnel to improve their capacity for bTB detection, diagnosis, and control measures. This will ensure timely and effective responses to suspected cases.
- Educate cattle owners, herders, and the general population about the risks associated with bTB transmission, and preventive measures, such as farm biosecurity, regular testing of cattle against bTB, proper hygiene practices, boiling/ pasteurization of milk for human consumption, to minimise the spread of bTB and other zoonotic diseases.
- Strengthen One Health collaboration by facilitating information sharing between animal and human health sectors, particularly regarding human TB and bovine TB cases, to enhance surveillance and response capabilities.
- Support research and development initiatives aimed at understanding the epidemiology and transmission dynamics of bTB in cattle and risk of human infection in Bhutan. Foster collaboration with international organizations and research institutions to share knowledge, resources, and best practices for bTB control and prevention.

# Develop and implement policies and regulations for the prevention, control, and management of bTB in Bhutan based on a clear understanding of the diseases’ epidemiology in cattle in the country. This may involve enforcing Livestock Act and Livestock Rules and Regulation of Bhutan, while incorporating updated strategies and guidelines for bTB control based on WOAH standards (56).

FIGURES
- Line 457: Figure 1: Could you please denote the four regions in Bhutan? Maybe use other colors? Could you extend the map to include the neighbouring countries? It could be useful to identify the region in the context provided in the first paragraph of Material and Methods.

Answer: We have made the changes in figure 1 and included the regions as well.

TABLES
- Table 2: Please include a column with the totals (%), the ORs and de 95% CI and include in the caption the analysis performed. You mention “Risk factors associated…”. Please change the title of the table as no evidence of statistically significant association between the evaluated risk factors and the outcome were found (p >0.05)

Answer: We have shown this information in the supplementary table 1.
